# Development of depressive symptoms during the COVID-19 crisis: the role of coping strategies and their change

**DOI:** 10.1186/s40359-025-02406-8

**Published:** 2025-03-14

**Authors:** Charikleia Lampraki, Daniela S. Jopp, Angélique Roquet, Adar Hoffman, Kim Uittenhove

**Affiliations:** 1 Department of Psychology, University of Geneva, Chemin de Pinchat 22, Carouge, Geneva, 1227 Switzerland; 2 Institute of Psychology, University of Lausanne, Lausanne, Switzerland; 3 Swiss Centre of Expertise in Life Course Research LIVES, Lausanne, Switzerland

**Keywords:** COVID-19, Depressive symptoms, Coping strategies, Pandemic, Age differences

## Abstract

**Background:**

Confronted with stressful circumstances, individuals use coping strategies to adapt. During the COVID-19 pandemic, individuals were threatened by an unprecedented health crisis, which governments tried to navigate with various imposed measures. Social distancing had massive negative consequences for mental health; yet studies also documented important interindividual differences, which may be related to differences in coping strategies. This study aims at identifying the most frequent coping responses, their change over time, as well as their possible role for adapting to the crisis.

**Methods:**

Our sample consisted of 732 individuals living in Switzerland (age range 18–81 years). An online three-wave questionnaire was administered during the second pandemic wave (i.e., October, November, and December 2020). We used bivariate latent growth modeling and multilevel modeling in order to investigate the development of depressive symptoms and the extent to which it related to the level and change in coping strategies, adjusting for sociodemographic characteristics.

**Results:**

Bivariate latent growth models showed that feeling depressed was related to lower use of seeking functional and emotional support, positive reappraisal and acceptance, and higher use of self-distracting. Moreover, results indicated that more change in depressive symptoms was related to less change in seeking functional support and positive reappraisal, and to more change in self-distracting. Regarding multilevel modeling, where all coping strategies were simultaneously included as predictors of depressive symptoms, a higher level of support seeking and positive reappraisal, and a lower level of self-distracting were related to fewer depressive symptoms. Over time, seeking support, positive reappraisal, acceptance, and self-distracting decreased, while depressive symptoms increased. Decreasing the use of positive reappraisal and increasing the use of self-distracting were related to increasing depressive symptoms. Younger aged individuals experienced significantly more depressive symptoms than their older age counterparts when they decreased the use of positive reappraisal.

**Conclusions:**

In conclusion, individuals used various coping strategies to adapt to the COVID-19-related life circumstances, but only some of them related to changes in depressing symptoms, possibly demonstrating a gap between the availability and use of coping strategies during the pandemic and their actual effect on mental health.

**Supplementary Information:**

The online version contains supplementary material available at 10.1186/s40359-025-02406-8.

## Background

Since its world-wide erratic spreading in early 2020, the COVID-19 pandemic has been an ongoing and continuously changing source of stress for people around the globe. Governmental suggestions and restrictions varied based on the propagation rate of the virus. In the absence of knowledge, potent medical treatment, or vaccination, social-distancing and sanitary measures were the most common governmental mitigation measures during the first year of the pandemic. Additionally, to prevent exponential growth of infections during the pandemic waves which would have led to collapsing medical structures, other measures were implemented such as obligatory home-office, operation of only first-necessity shops, and generalized lockdowns. During the second wave of the pandemic in the fall/winter of 2020, the large majority of countries went into lockdown to prevent infection numbers to aberrate; while there were also notable increases of COVID-19 infections including increased mortality, Switzerland opted for lighter mitigation measures, including home office, closing of bars, restaurants, non-essential shops, and leisure locations, providing the opportunity to study the consequences of the COVID-19 pandemic in a different, less restrictive context than in most European countries.

Along with the health-related stress of fearing a COVID-19 infection [1], individuals had to adapt to these unprecedented, imposed, and constantly changing conditions, which have been found to have notable negative consequences for their mental health [2, 3], such as elevated anxiety [4–6], social and emotional loneliness [7–9], isolation [10, 11], and depressive symptoms [4, 6, 12, 13], among others. A possible explanation for the decrease in mental health during the pandemic may be that many of the factors known to be responsible for poor mental health became more prevalent (e.g., unemployment, reduced mobility, limited social contacts), leading to increased levels of depressive symptomatology, with women and younger aged individuals being more affected [14]. Moreover, Daniali and colleagues [6] found that depression increased the most during the pandemic in comparison to anxiety and stress across the globe and that younger and student populations were mostly affected. Above and beyond usual prevalence rates, an additional 53.2 million of individuals have developed depressive disorders during the first year of the pandemic; this increase highlights the severe mental health consequences of the global burden associated to COVID-19 [14]. Moreover, it demonstrates the need to investigate the cognitive and behavioral mechanisms which have helped individuals to adapt to the hardships of the pandemic, or may, in the absence of any experience with similar previous situations, even have led to an increase in depressive symptomatology. Having a closer look at potentially underlying mechanisms is also important in the light of the substantial interindividual differences in mental health outcomes which have been observed. Because despite the documented negative mental health consequences, not everyone became depressed, and an important question is why this was the case.

The response and adaptation to stressful circumstances differ among individuals [15]. Coping is defined by Folkman and Lazarus [16] as the behavioral or cognitive response to manage internal or external stressors that may increase anxiety or other adverse psychological outcomes. Researchers have categorized coping strategies in different ways: problem-focused vs. emotion-focused coping, engagement vs. disengagement, approach vs. avoidance coping, primary- and secondary-control, and assimilative vs. accommodative coping [17–20] (for an in-depth discussion, see [21]). Some of the coping strategies are focusing on resolving a specific problem (problem-centered strategies, e.g., problem solving, seeking social or emotional support) while others are focusing on changing aspects of the person to better live with the problem, and represent therefore self-centered strategies (e.g., acceptance, positive reappraisal, self-distracting).

The ability to use the most adaptive coping strategy for a particular stressor can have short-term and long-term consequences for mental health [22]. Which coping strategy is useful depends on the nature of the stressor, and there is evidence that strategy use changes with age [23, 24]. For instance, Brennan and colleagues [23] found that, overall, the use of coping strategies declined with age, but the rate of decline was related to stressors’ appraisal, their nature, and available resources. Some coping strategies seem appropriate for dealing with the potential loss of resources (i.e., seeking functional or emotional support), while others are more effective for facing the accumulation of lost resources (acceptance, positive reappraisal [22]).

In the context of the pandemic, individuals faced actual losses such as the reduction of social interactions, the cessation of sports activities, and the rendering of services, as well as fear regarding the future progression of the pandemic and their everyday life consequence. Given the novelty of this health crisis and its consequences for everyday life, many individuals may have felt unable to find appropriate coping responses, or their coping efforts did not help in this context [25]. So far, studies have addressed the coping responses and their effects during the COVID-19 pandemic in various countries around the world [5, 25–29]. For instance, in a sample of healthcare workers, avoiding thinking of the pandemic, not being sure of how to cope, or struggling to cope with new difficulties were related to higher anxiety and depression [5]. Moreover, Fluharty et al. [28] found that individuals who used more active problem solving, support seeking, and avoidant coping presented more depressive symptoms, while receiving social support was protective against depressive symptomatology during the pandemic. Finding evidence that active problem-solving and support seeking are associated with negative mental health is rather unusual and may be pandemic-specific, because these strategies are known to be associated with better adaptation outcomes. Specifically, it is likely that the consequences of COVID-19 affected all central life-domains simultaneously and many individuals experienced the pandemic as very unpredictable and overwhelming. Therefore, trying to adapt using problem-centered strategies may have led to worse rather than better outcomes, such as increased stress and depressive symptoms. Regarding support seeking, it may be that the requests for help were not leading to actual support: Approached individuals struggled themselves with the pandemic and were unable to provide help.

It is important to also consider the coping response with regard to lifespan differences. Older aged individuals tend to use more self-centered accommodative strategies (e.g., positive reappraisal) in comparison to their younger counterparts, who use more frequently problem-centered assimilative coping (e.g., active problem solving; [15]). This use of strategies was also evident during the COVID-19 pandemic: Older aged individuals used more self-centered strategies, such as acceptance and self-distracting, than problem-focused [30]. Nevertheless, despite individual preferences and age-associated differences, research shows that individuals can use different coping strategies individually or in combination when needed. However, research is limited regarding which coping strategies individuals employed to cope with the advancement of the pandemic (e.g., new pandemic waves), whether any changes occurred during this time and how they related to depressive symptoms.

In the present study, we aimed at investigating the use of coping strategies and their change over a period of three months during the second pandemic wave (i.e., October, November, December 2020), and how their trajectories were associated with the trajectory of depressive symptoms over time, using a lifespan sample. Specifically, we tested first how change in each coping strategy (i.e., seeking functional support, seeking emotional support, positive reappraisal, acceptance, and self-distracting) separately related to change in depressive symptoms and then, which of the coping strategies (tested concurrently) related more strongly to intraindividual change and interindividual differences in depressive symptoms while controlling for a set of sociodemographic variables (i.e., age, gender, marital status, living alone, employment status, and education years). Finally, we tested whether age played a moderating role on the link between coping strategies and depressive symptoms.

We expected that (H1), as problem-centered strategies (e.g., seeking support) were limited by imposed restrictions (e.g., social distancing measures) and loss of resources (e.g., financial resources), individuals likely turned more to self-centered coping styles (e.g., positive reappraisal) during the advancement of the pandemic. In addition, we hypothesized that (H2) initially engaging more in support seeking strategies (e.g., seeking functional or emotional support) as well as avoidant coping strategies (e.g., self-distracting), and less in self-centered coping strategies (e.g., positive reappraisal and acceptance) would be related to higher depressive symptoms. Similarly, we expected in line with prior findings [31] that (H3) increasing the use of support seeking and self-distracting and decreasing the use of positive reappraisal and acceptance would relate to increasing depressive symptoms. Moreover, we hypothesized that (H4) individuals who on average used more support seeking strategies and self-distracting would have higher depressive symptoms, while those who used self-centered strategies would have lower depressive symptoms. Regarding how changes in coping strategies related to the development of depressive symptoms, we expected (H5) that more change in support seeking and self-distracting and less change in self-centered strategies would relate to a steeper increase in depressive symptoms. Finally, in line with developmental theories expecting better emotion-regulation (e.g [32]), and previous work on an age-related shift in coping responses towards more accommodative strategies (e.g [17]), we hypothesized that (H6) the change in depressive symptoms would differ between younger and older aged individuals with regards to changes in the use of self-centered coping strategies. Given that these self-centered coping strategies depend on internal resources, such as motivational processes [33], older adults’ higher competence to deal with “unchangeable” stressful situations may have protected them from experiencing an increase in depressive symptoms, in comparison to younger aged adults.

## Methods

### Sample and procedure

We conducted a longitudinal study in Switzerland during the second wave of the COVID-19 pandemic to identify its impact on mental health outcomes across adulthood and older age. The study was conducted in French language, one of the official languages of Switzerland. Data collection included three study waves between October and December 2020, assessed with a one-month interval. In Switzerland, October 2020 was the month before the rapid increase of infections, representing the local start of the second pandemic wave, characterized by exponentially increasing infection cases (6.92/100000 inhabitants on the 01.10.2020 to 49.1/100000 inhabitants on the 01.12.2020) and high mortality rates (0.06/100000 inhabitants on the 01.10.2020 to 1.01/100000 inhabitants on the 01.12.2020; Federal Office of Public Health, 2022). The government and regional authorities reacted with increasing public measures, leading to a “semi-lockdown”: While sanitary and social recommendations were limited to basic hygiene and physical distancing in public spaces in October, more severe restrictions and recommendations were set in place in November, including distance-learning in universities, and limitations on the number of people in social gatherings. In December 2020, the government ordered obligatory home-office and closing of restaurants, bars, sport utilities and non-essential shops. Given the increase in suggested or imposed measures to delay the spread of the virus, this study allowed us to investigate the status and longitudinal changes of coping strategies and depressive symptoms and how the former could relate to changes in the latter in these unprecedented conditions. The sample consisted of *N* = 732 individuals, aged between 18 and 81 years (M_age_ = 31.52). University students comprised 47% and their entourage (family, friends, acquaintances, social network) 53% of the sample. All participants had reached the Swiss legal age of majority (i.e., 18 years old) and provided informed consents before filling out the online questionnaire. The study was not pre-registered but was included in the ethics request document and received the approval of the ethics commission for the social and political sciences of the University of Lausanne.

### Measures

#### Sociodemographic variables

The sociodemographic variables included *age*, *gender* (0 = *men*, 1 = *women*), *marital status* (0 = *non-married*, 1 = *married*), *living alone* (0 = *no*, 1 = *yes*), *being employed* (0 = *no*, 1 = *yes*), and *education years*. *Time* represented the study wave and ranged from 0 = *study wave one (October)* to 2 = *study wave three (December)*.

#### Coping strategies

Five coping strategies from the Jopp and colleagues [34] Multi-dimensional Coping Inventory (MDCI) were selected as most relevant for adapting to a prolonged, collective, and stressful situation such as the COVID-19 pandemic. We assessed the five most relevant dimensions with a total of 21 items: *Seeking functional support* (4 items, e.g., “When things get difficult, do you ask for help to keep up with your daily activities?”; Cronbach’s *α*: 0.88-0.90), *seeking emotional support* (4 items, e.g., “When things are tough, do you look for sympathy?”; Cronbach’s *α*: 0.85-0.86), *positive reappraisal* (4 items, e.g., “When things are tough, do you tend to see the silver lining?”; Cronbach’s *α*: 0.83-0.87), *acceptance* (4 items, e.g., “When things get tough, do you try to accept the situation?”; Cronbach’s *α*: 0.81-0.85), and *distracting* (5 items, e.g., “Do you do things to keep your mind off a problem, such as watching TV, sleeping, or shopping?”; Cronbach’s *α*: 0.87-0.91). The answering options assessed the frequency of use of coping strategies (0 = *not at all*, 1 = *a little*, 2 = *moderate amount*, 3 = *quite a bit*, and 4 = *very often)*. Mean composite scores were created for each coping strategy with higher values suggesting more frequent use of the specific strategy.

#### Depressive symptoms

Depressive symptoms were measured with the short CES-D scale (Center for Epidemiological Studies Depression Scale [35], 15-item version from Meyer and Hautzinger [36]). Participants were asked to indicate how often they experienced certain symptoms in the past week (example items: “I felt that I could not shake off the blues even with help from my family or friends”, “I felt depressed”). The answering format ranged from 1 = *rarely/not at all (less than once a day)* to 4 = *most of the time/all the time (for 5 to 7 days)*. A mean composite score was created with higher values indicating more depressive symptoms (Cronbach’s *α*: 0.80-0.84).

### Analytical strategy

First, we calculated the descriptive statistics for the overall sample and for each study wave, as well as pooled-across-waves correlations between all central study variables. For the main analysis, we first tested bivariate latent growth-curve models (LGM) to investigate the longitudinal trajectories of the five coping strategies (e.g., seeking functional support) and depressive symptoms, which were then followed by a multilevel linear model with depressive symptoms as the dependent variable and all the coping strategies as independent variables. The chosen methodological approach offers the advantage of modelling first the structure and growth of two variables concurrently (i.e., five bivariate LGMs with depressive symptoms and each coping strategy separately), while the multilevel model allows for simpler model specification by including the mean and change around the mean of multiple variables (i.e., all the coping strategies together) for the investigation of between-subjects differences and within-subjects variation in a single outcome (i.e., depressive symptoms). Combining these two powerful statistical approaches, first the bivariate latent growth models and then the multilevel model, allows to draw conclusions about the trajectories of the coping strategies and depressive symptoms but also to identify which coping strategies were more important for depressive symptoms, with regard to their mean level and change. Sociodemographic information included age, gender, being married, living alone, being employed, and educational years, which served as control variables.

For the bivariate LGMs, we estimated the linear growth as well as all covariances between intercepts and slopes of five sets of variables: (1) seeking functional support and depressive symptoms, (2) seeking emotional support and depressive symptoms, (3) positive reappraisal and depressive symptoms, (4) acceptance and depressive symptoms, and (5) self-distracting and depressive symptoms. The variances and intercepts were freely estimated. Significant variances indicated that the changes in depressive symptoms and/or coping strategies varied over time and differed with regards to the initial level at the start of the study (wave 1). In all models, we fitted linear slope loadings of [0, 1, 2] for waves 1, 2, and 3, respectively, and intercept loadings of [1, 1, 1] [37]. Using the FIML function in Lavaan, we accounted for missing data and used all available information (*N* = 736). Conducting the same analyses with listwise deletion (*N* = 457, complete data only) we obtained similar results. Model fit was evaluated based on the χ^2^-test, the Comparative Fit Index (CFI), the Tucker Lewis Index (TLI), the Standardized Root Mean Square Residual (SRMR), and the Root Mean Square Error of Approximation (RMSEA) with 90% confidence intervals. Following the recommendations by Hu and Bentler [38], good data fit was confirmed when CFI and TLI were ≥ 0.95, SRMR was ≤ 0.08 and RMSEA was ≤ 0.06. The presented estimates for all LGMs are unstandardized.

For the multilevel model, we centered the time-varying variables (i.e., coping strategies) to facilitate the interpretability of the within-subjects variation and to gain more stable estimates [39, 40]. We also included the across-waves person-mean of the coping strategies to investigate between-subjects differences. All control variables were measured in wave one and included in the model as time-invariant non-centered factors. Given that age did not vary significantly over the one-month intervals across the study, age was also considered as time-invariant. We present the final and most parsimonious model (i.e., the one with the best fit) which tested fixed and random effects, as well as an interaction term, and estimates are unstandardized. The fit of the model was tested with the Akaike’s Information Criterion (AIC) and the − 2 log likelihood (-2LL) fit indices. The procedure leading to the presented final model was as follows: First, we tested a fully unconditional model, with no predictors to identify to what extent between-subjects differences and within-subjects variation were attributed to the hierarchical clustering of the data. Given that the results of the unconditional model justified the use of multilevel modelling, we added the control variables and the fixed effects of the coping strategies into the model. This model was then further complemented by adding interaction effects between the coping strategies and age (one after the other), retaining only the interaction effects that improved the model fit. Finally, we added the random parameters for the coping strategies one by one in separate models, resulting in the final model which included only those that improved the overall fit. All models were tested using Maximum Likelihood estimation method, which also handles missing data. The analyses were performed with R [41] and the nlme package [42] for multilevel modeling, while the LGM analyses were conducted with the Lavaan package [43].

## Results

Descriptive statistics and Pearson correlations are presented in Tables 1 and 2.

**Table 1:** Descriptives of study variables

	Wave 1 (*n* = 732) M or *N* (SD or %)	Wave 2 (*n* = 650) M or *N* (SD or %)	Wave 3 (*n* = 522) M or *N* (SD or %)
Age	31.53 (13.58)	-	-
< 25 years old	355 (49%)		
25–49 years old	251 (34%)		
> 50 years old	122 (17%)		
Gender (Women)	489 (66%)	-	-
Married (Yes)	141 (19%)	-	-
Living alone (Yes)	91 (12%)	-	-
Employed (Yes)	276 (38%)	-	-
Education years	13.25 (2.72)	-	-
Seek functional support	2.12 (0.95)	2.08 (0.92)	2.07 (0.95)
Seek emotional support	2.24 (1.00)	2.14 (0.98)	2.09 (0.97)
Positive reappraisal	2.16 (0.90)	2.17 (0.88)	2.09 (0.88)
Acceptance	2.31 (0.74)	2.29 (0.73)	2.20 (0.76)
Self-distracting	2.06 (1.00)	2.07 (0.97)	1.98 (0.96)
Depression	1.75 (0.55)	1.85 (0.57)	1.89 (0.59)

**Table 2:** Correlations of study variables (pooled-Across-Waves)

	1	2	3	4	5	6	7	8	9	10	11
1. Age	1										
2. Gender (Women = 1)	− 0.10**	1									
3. Married (Yes = 1)	0.63**	− 0.05*	1								
4. Living alone (Yes = 1)	0.01	− 0.09**	− 0.18**	1							
5. Employed (Yes = 1)	0.42**	− 0.21**	0.32**	0.08**	1						
6. Years of education	0.30**	− 0.19**	0.19**	0.06**	0.44**	1					
7. Seek functional support	− 0.11**	0.22**	− 0.07**	− 0.08**	− 0.01	0.01	1				
8. Seek emotional support	− 0.15**	0.27**	− 0.11**	− 0.06**	− 0.05*	0.00	0.81**	1			
9. Positive reappraisal	0.14**	− 0.11**	0.08**	0.03	0.16**	0.10**	0.20**	0.21**	1		
10. Acceptance	0.14**	− 0.06**	0.12**	− 0.00	0.15**	0.05*	0.15**	0.17**	0.52**	1	
11.Self-distracting	− 0.27**	0.13**	− 0.24**	0.05*	− 0.13**	− 0.05*	0.16**	0.20**	− 0.07**	− 0.06**	1
12. Depressive Symptoms	− 0.25*	0.13**	− 0.19**	0.08**	− 0.27**	− 0.12**	− 0.13**	− 0.07**	− 0.41**	− 0.26**	0.26**

Note. * *p* <.05. ** *p* <.01.

The bivariate latent growth curve models investigated the extent to which the trajectories of the coping strategies related to the trajectory of depressive symptoms, in five separate models. Table 3 presents the model parameters and fit indices for each bivariate LGM. All models showed a good fit to the data as indicated by the fit indices. Across all models, depressive symptoms increased over time, as evidenced by the mean value of the slope in each model (e.g., *µ*_*s*​_ = 0.08, *p* <.001 in Model 1). Moreover, as depicted in Table 3, the intercept and slope variances in depressive symptoms were significant, indicating that the initial level and the rate of change varied among participants; this finding was similar across all bivariate LGMs. In addition, all coping strategies decreased over time (Table 3, e.g., *µ*_*s* ​_= − 0.08, *p* <.001 in Model 2 for seeking emotional support), but the rate of change differed only for positive reappraisal (e.g., *σ*^*2*^_*s*_ = 0.04, *p* <.05), indicating that the decrease was different between individuals for this specific strategy. Regarding the initial level of coping strategies, we found significant intercept variances (e.g., *σ*^*2*^_*i*_ = 0.65, *p* <.001 in model 5 for self-distracting) indicating that individuals had different initial levels in coping strategies.

In the first model, we tested the relationship between seeking functional support and depressive symptoms. We found a significant negative intercept-intercept covariance showing that when individuals had a low initial level of seeking functional support, they experienced a higher level of depressive symptoms at baseline (*σ*_*ii*_ = − 0.09, *p* <.001). Moreover, the intercept of seeking functional support was positively related to the slope of depressive symptoms, indicating that when the initial level of seeking functional support was at the highest then the depressive symptoms decreased at the fastest rate (*σ*_*is*​_ = 0.03, *p* <.01). We also found a significant slope-slope negative covariance (*σ*_*ss*_ = − 0.01, *p* <.05) suggesting that a steeper decrease in seeking functional support was related to a steeper increase in depressive symptoms over time.

Next, we tested the relationship between seeking emotional support and depressive symptoms. Results indicated that a low initial level in seeking emotional support related to a high baseline level in depressive symptoms, reflected in the significant negative intercept-intercept covariance (*σ*_*ii*_ = − 0.07, *p* <.01). The intercept of seeking emotional support and the slope of depressive symptoms were positively related (*σ*_*is*​_ = 0.02, *p* <.05), indicating that individuals who had the highest initial level in seeking emotional support experienced a steeper decline in depressive symptoms.

Regarding the relationship between positive reappraisal and depressive symptoms we found that a low initial level in positive reappraisal related to a high initial level in depressive symptoms (*σ*_*ii*_= -20, *p* <.001), as indicated by the significant negative intercept-intercept covariance. A negative slope-slope covariance (*σ*_*ss*_ = − 0.02, *p* <.001) was found, suggesting that a steeper decline in positive reappraisal related to a steeper increase in depressive symptoms, over time.

Next, we tested the relationship between acceptance and depressive symptoms and found a significant negative intercept-intercept covariance (*σ*_*ii*_ = − 0.09, *p* <.001), suggesting that a low initial level in acceptance related to a high initial level in depressive symptoms.

Finally, the last LGM tested the relationship between self-distracting and depressive symptoms. We found a significant and positive intercept-intercept covariance (*σ*_*ii*_ = 0.14, *p* <.001), indicating that a high initial level in self-distracting related to a high initial level in depressive symptoms. Moreover, results showed that when individuals had the highest initial level in depressive symptoms, they experienced a slower decline in self-distracting, as indicated by the significant negative intercept-slope covariance (*σ*_*is*_ = − 0.02, *p* <.05). A steeper increase in self-distracting was, finally, related to a steeper increase in depressive symptoms over time, as shown by the significant positive slope-slope covariance (*σ*_*ss*_ = 0.01, *p* <.05). Using multilevel modeling, we then tested which factors predicted between-subjects differences and within-subjects changes in depressive symptoms when including all the coping strategies simultaneously in the model. The first fully unconditional model (no predictors included) had an Intraclass Correlation Coefficient of *ρ* = 0.64, indicating that 64% of the trajectories of change in depressive symptoms varied across individuals. Therefore, more complex multilevel models were appropriate to investigate the between-subjects differences and the within-subjects change.

**Table 3:** Model parameters and fit indices of the bivariate latent growth models of depression and coping strategies

	Intercept		Slope		Intercept– Slope	Intercept (Depressive symptoms)– Intercept (Coping Strategy)		Slope (Depressive symptoms)– Slope (Coping Strategy)			Intercept (Depressive symptoms)– Slope (Coping Strategy)		Intercept (Coping Strategy)– Slope (Depressive symptoms)				
	(µ_i_)	(σ^2^_i_)	(µ_s_)	(σ^2^_s_)	(σ_is_)	(σ_ii_)		(σ_ss_)			(σ_is_)		(σ_is_)				
*Model 1*	χ^2^ = 10.607; *p* =.16; df = 7; CFI = 0.998 ; TLI = 0.995; RMSEA = 0.031, 90%CI = (0.000, 0.066); SRMR = 0.010																
Depressive symptoms	1.76^***^	0.22^***^	0.08^***^	0.02^*^	− 0.01		− 0.09^***^		− 0.01^*^	0.002		0.03^**^					
Seeking functional support	2.12^***^	0.66^***^	− 0.03^+^	0.01	− 0.02												
*Model 2*	χ^2^ = 10.669; *p* =.16; df = 7; CFI = 0.998 ; TLI = 0.996; RMSEA = 0.029, 90%CI = (0.000, 0.064); SRMR = 0.011																
Depressive symptoms	1.76^***^	0.22^***^	0.08^***^	0.02^*^	− 0.01		− 0.07^**^		− 0.01	0.01		0.02^*^					
Seeking emotional support	2.23^***^	0.78^***^	− 0.08^***^	0.03	− 0.04												
*Model 3*	χ^2^ = 16.560; *p* =.02; df = 7; CFI = 0.995 ; TLI = 0.989; RMSEA = 0.048, 90%CI = (0.016, 0.080); SRMR = 0.014																
Depressive symptoms	1.76^***^	0.22^***^	0.07^***^	0.02^+^	− 0.01		− 0.20^***^		− 0.02^***^	0.01		0.02^+^					
Positive reappraisal	2.18^***^	0.61^***^	− 0.03^*^	0.04^*^	− 0.04^+^												
*Model 4*	χ^2^ = 5.828; *p* =.56; df = 7; CFI = 0.995 ; TLI = 0.989; RMSEA = 0.047, 90%CI = (0.000, 0.082); SRMR = 0.012																
Depressive symptoms	1.76^***^	0.23^***^	0.08^***^	0.02^*^	− 0.01		− 0.09^***^		− 0.01	0.000		0.002					
Acceptance	2.33^***^	0.30^***^	− 0.06^***^	0.01	0.01												
*Model 5*	χ^2^ = 17.145; *p* =.02; df = 7; CFI = 0.992 ; TLI = 0.983; RMSEA = 0.052, 90%CI = (0.022, 0.084); SRMR = 0.016																
Depressive symptoms	1.76^***^	0.22^***^	0.08^***^	0.02^*^	− 0.01		0.14^***^		0.01^*^	− 0.02^*^		− 0.002					
Self-distracting	2.07^***^	0.65^***^	− 0.03^+^	0.04	− 0.06												

Note: *µ* indicates mean. *σ*^*2*^ indicates variance. *σ* indicates covariance. i refers to intercept and s refers to slope. ^+^*p* <.10; ^*^p <.05; ^***^p <.001.

**Fig. 1:**
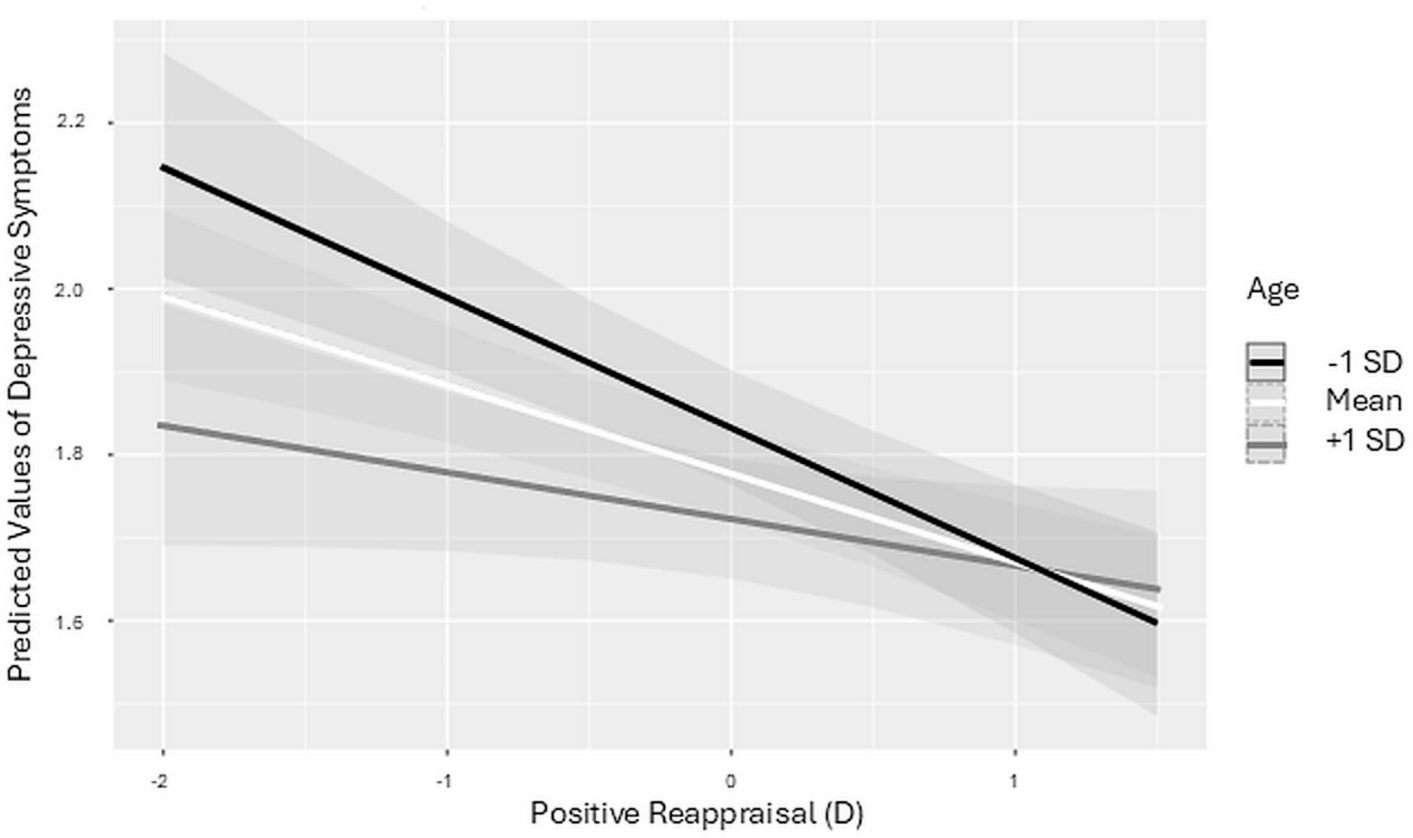
Graphical representation of the interaction between positive reappraisal (change) and age on predicted values of depressive symptoms

The most parsimonious model is presented in Table 4. As the two variables assessing seeking functional support and seeking emotional support were highly correlated (*r* =.81), we calculated a score of support seeking combining the two variables for the multilevel analysis (for separate models see Appendix A). For the between-subjects differences our results showed that with time individuals experienced more depressive symptoms (*B* = 0.07; 95%CI: [0.05–0.09]). In addition, younger individuals (*B* = − 0.004; 95%CI: [-0.01– -0.001]), women (*B* = 0.09; 95%CI: [0.02–0.16]), people living alone (*B* = 0.14; 95%CI: [0.04–0.24]) and those without employment (*B* = − 0.18; 95%CI: [-0.26– -0.10]) felt more depressed. Moreover, individuals who overall sought more support (*B* = − 0.08; 95%CI: [-0.12– -0.04]), engaged more in positive reappraisal (*B* = − 0.21; 95%CI: [-0.26– -0.17]), and less in self-distracting (*B* = 0.14; 95%CI: [0.10–0.18]) experienced on average fewer depressive symptoms. Regarding the within-subjects effects, a one unit decrease in support seeking and positive reappraisal and a one unit increase in self-distracting related to an increase of depressive symptoms equal to *B* = − 0.08 (95%CI: [-0.13– -0.02]), *B* = − 0.22 (95%CI: [-0.33– -0.12]) and *B* = 0.06 (95%CI: [0.02–0.10]), respectively. The significant interaction effect (*B* = 0.004; 95%CI: [0.001–0.01]; Fig. 1), confirmed an interplay between change in positive reappraisal and age with respect to depressive symptoms: Specifically, when individuals increased the use of positive reappraisal, their levels of depressive symptoms decreased regardless of their age. However, when individuals reduced the use of positive reappraisal the effect on depressive symptoms differed between younger and older adults: Reduction in positive reappraisal made younger individuals significantly more depressed compared to their older-aged counterparts.

**Table 4:** Multilevel Model effects of between- and within-subject covariates of depressive symptoms

**Fixed Between Subjects Effects**	Estimates	CI
Time	**0.07**	**0.05–0.09**
Age	**-0.004**	**-0.01 – -0.001**
Gender (Women = 1)	**0.09**	**0.02–0.16**
Married (Yes = 1)	-0.01	-0.11–0.10
Living alone (Yes = 1)	**0.14**	**0.04–0.24**
Employed (Yes = 1)	**-0.18**	**-0.26 – -0.10**
Education years	0.01	-0.00–0.02
Seek support (mean)	**-0.08**	**-0.12 – -0.04**
Positive reappraisal (mean)	**-0.21**	**-0.26 – -0.17**
Acceptance (mean)	-0.05	-0.11–0.01
Self-distracting (mean)	**0.14**	**0.10–0.18**
**Fixed Within Subject Effects**		
Seek support (change)	**-0.08**	**-0.13 – -0.02**
Positive reappraisal (change)	**-0.22**	**-0.33 – -0.12**
Acceptance (change)	0.03	-0.01–0.07
Self-distracting (change)	**0.06**	**0.02–0.10**
Positive reappraisal (change)* Age	**0.004**	**0.00–0.01**
**Random Effects**		
Residual Variance	**0.08**	**0.27–0.31**
Intercept	**0.13**	**0.33–0.38**
Seeking support slope	**0.04**	**0.11–0.25**
Self-distracting slope	**0.04**	**0.16–0.27**
Intercept * Seeking functional support slope	-0.34	-0.52–0.04
Intercept * Self-distracting Slope	0.18	-0.04–0.36
ICC	0.64	
N	696	
Observations	1762	
Marginal R^2^ / Conditional R^2^	0.302 / 0.748	

Considering the random effects, the within-subjects random variance (*B* = 0.08; 95%CI: [0.27–0.31]) and the random intercept (*B* = 0.13; 95%CI: [0.33–0.38]) were significantly different from zero. These results indicate that there was significant variability in depressive symptoms within each individual over time and significant differences between individuals with regard to their baseline level, after accounting for all other variables in the model. The random slopes of seeking support (*B* = 0.04; 95%CI: [0.11–0.25]) and self-distracting (*B* = 0.04; 95%CI: [0.16–0.27]) also varied significantly, suggesting that their rates of change differed across individuals. The covariances between the intercept and the slopes of seeking support (*B* = − 0.34; 95%CI: [-0.52–0.04]) and self-distracting (*B* = 0.18; 95%CI: [-0.04–0.36]) were not significant, indicating that the rate of change in depressive symptoms was not related to the change in these two coping strategies.

## Discussion

This study investigated the concurrent trajectories of coping strategies and depressive symptoms during the second pandemic wave of COVID-19 in Switzerland. Moreover, it examined the extent to which interindividual differences and intraindividual change in coping strategies were associated to differences in the level and change (from the mean) of depressive symptoms. Findings indicated that individuals reduced the use of problem-centered and self-centered coping strategies over time. Concurrently, depressive symptoms increased. The rate of change in specific coping strategies was associated with the rate of change in depressive symptoms. Specifically, a steeper decrease in the use of functional support and positive reappraisal and a steeper increase in self-distracting was related to a steeper increase in depressive symptoms. Regarding the multilevel associations when including all the coping strategies as predictors of depressive symptoms we found that: (a) higher support seeking and positive reappraisal and lower levels of self-distracting related to fewer depressive symptoms; (b) decreasing support seeking and positive reappraisal and increasing self-distracting was related to increasing depressive symptoms; (c) the rate of change in depressive symptoms, seeking support and self-distracting varied significantly between individuals; d) a decrease in positive reappraisal was associated with a steeper increase in depressive symptoms, and this effect was stronger for young compared to older individuals. We will discuss these four key findings in the following paragraphs separately.

### Coping strategies and depressive symptoms changed significantly over time

Our study covered the month right before the start of the second COVID-19 pandemic wave in Switzerland and the two months that followed. We found that over time all coping strategies decreased to a smaller or greater extent, suggesting that individuals had difficulty maintaining their coping efforts being confronted with the next pandemic wave. Specifically, seeking functional and/or emotional support, positive reappraisal, acceptance, and self-distracting decreased (or tended to decrease) over time, as evidenced by the findings in both analytical approaches. These findings are partly in contrast to our first hypothesis (H1) where we expected an increase of person-centered strategies, such as acceptance and positive reappraisal.

With increasing restrictions by the government and regional authorities, and augmenting infection and mortality (i.e., during the second and third study waves), individuals may have become more hesitant or less efficient in asking for functional and/or emotional support. Home-office and restricted social contacts may have limited their interpersonal interactions [8], making it difficult to ask and receive help for practical as well as for emotional issues. Moreover, we observed a significant decrease in the self-centered coping strategies, such as positive reappraisal and acceptance, and in self-distracting, that represents some type of more avoidant coping. However, it is of note that acceptance remained their favorite coping strategy, whereas self-distracting was their least preferred. These findings may suggest that individuals felt more and more tired of the pandemic. During the first-wave lockdown, many people stressed the positive effects brought by the pandemic, such as learning new things [44], showing positive reappraisal. However, keeping up this coping strategy became challenging, as individuals found it increasingly difficult to find positive sides or come to peace with the issues they faced, as even for such internal strategies, some energy and effort was needed, yet people were probably already too worn out at this moment. Our findings further showed that, during the second pandemic wave, individuals also reduced self-distracting; while self-distracting does not promote the resolution of an existing problem it may facilitate dealing with emotions, and it has proved particularly relevant in the context of the pandemic where the actual problem could not be fixed [5]. Although being an easily accessible and low energy consuming coping strategy, use of self-distracting became less attractive as did all the other strategies. The reduction in use of coping strategies may suggest that individuals followed a trial-error approach to find the strategies that fit them better, illustrating an overall coping fatigue.

As one could expect, when the use of coping strategies decreased, depressive symptoms increased in parallel, which is in line with previous studies demonstrating a global increase of depression [6, 13]. While the overall sample did not show very high depressive symptoms, levels still increased. In sum, these findings suggest that individuals may have felt incapable of taking action to resolve the issues brought by the new pandemic wave and its consequences, reducing the use of their coping strategies, and at the same time they experienced worse mental health.

### Initial and person-average levels of coping strategies related to depressive symptoms

Regarding the relationship between levels of coping strategies and levels of depressive symptoms we found the following results: Having initially low levels of seeking support (functional or emotional), positive reappraisal and acceptance, as well as high levels of self-distracting was related to high initial levels of depressive symptoms, which partly confirmed our second hypothesis (H2). Similarly, when testing how the person-mean in coping strategies related to the level in depressive symptoms, we found that low support seeking, low positive reappraisal and high self-distracting were associated with more depressive symptoms. These findings partly confirm our fourth hypothesis (H4), as only high use (personal average) of self-distracting was related to more depressive symptoms and not high use of support seeking. Moreover, the results are in line with previous research indicating that seeking support and reevaluating an event as less stressful, relate to better mental health [45]. However, our findings also contrast previous research which found that higher support seeking related to more depressive symptoms during the COVID-19 pandemic [28]. This deviation from previous findings from the pandemic period may be related to the fact that our study was conducted in Switzerland, in a later stage of the pandemic, and with less restrictive measures compared to other countries. Self-distracting has also been documented to relate to more depressive symptoms [28, 45] and our finding also supports our second hypothesis (H2).

It is of note that seeking emotional support and acceptance were negatively associated to depressive symptoms’ levels, indicating that using more emotional support and acceptance strategies were linked to fewer depressive symptoms. In the multilevel model, however, where all coping strategies were included simultaneously as predictors in the model, acceptance did not show a significant mean-level association to depressive symptoms’ average levels, in contrast to our hypotheses (H4), indicating that regardless of the high frequency of use of acceptance, depressive symptoms were similar across individuals. This finding partly contrasts past research conducted before COVID-19, showing that individuals who used acceptance had better mental health outcomes [46, 47]. A possible explanation may be associated to the specific pandemic situation: accepting the new life situation may have been unfeasible, because of the constantly changing regulations and the commonly felt uncertainty about the future [48]. Taken together, the slightly different findings of the two methodological approaches may be related to the fact that we included all the coping strategies simultaneously in the multilevel model, limiting the variance explained by acceptance. It may, however, also be related to the fact that in the bivariate growth curve models we report findings regarding the initial level of the outcome and the predictor instead of the person-mean level across the three measurement points, which was used in the multilevel model.

### A faster increase in depressive symptoms related to the rate of change in the use of specific coping strategies

Regarding changes in coping strategies and their effects on depressive symptoms, we found that an increase in positive reappraisal and seeking support and a decrease in self-distracting related to a decrease in depressive symptoms. Additionally, we found that when the increase in use of positive reappraisal and of seeking functional support was steeper, then the depressive symptoms decreased at a faster rate. Similarly, when the increase in self-distracting was steeper, depressive symptoms also increased at a faster rate. Therefore, evidence from both the latent growth models and the multilevel model partly confirmed hypotheses three (H3) and five (H5). It is of note, however, that differences in findings are associated with the focus and set-up of the models, as the latent growth models inform about the parallel trajectories of the coping strategies and the depressive symptoms, while the multilevel models highlight how the deviation from the personal overall average may relate to changes in depressive symptoms. Moreover, changes in acceptance did not relate to changes in depressive symptoms, even though its levels significantly decreased with time. These findings show that, during the second pandemic wave, specific self-centered and problem-centered coping strategies played an important role in maintaining (or reducing) mental health. The fact that acceptance was not related to depressive symptoms, even though it is a self-centered strategy that was most often used, may be related to the fact that COVID-19 was not a distinct critical life event in one’s life course but rather an ongoing, relentlessly changing stressful period that was affecting (and still is) collectively the society. Therefore, it was not considered as a new difficult life circumstance that one must accept and move on with life, but a constantly evolving threat that could implicate new challenges from one day to another, making acceptance as a strategy more difficult to apply and having fewer positive effects as usually observed.

### Younger individuals experienced more depressive symptoms with more change in positive reappraisal

Our results indicate that age moderated the effect between the change in the use of positive reappraisal and depressive symptoms, confirming our sixth hypothesis (H6). Specifically, when individuals experienced more change in positive reappraisal, they felt less depressed regardless of whether they were younger or older in age. However, when they changed less regarding the use of positive reappraisal, we found significant differences in their depressive symptoms: Younger aged individuals felt more depressed than their older aged counterparts. These findings thus suggest that younger aged individuals who did not try to re-evaluate positively the stressful situation experienced more depressive symptoms. In line with previous research showing that older aged individuals tend to use more emotion regulation [32] and accommodative strategies to face challenges [15], older adults may be more accustomed to positively reevaluating stressful life circumstances, than their younger aged counterparts.

### Limitations

Despite the new insights regarding coping strategies and their relation to depressive symptoms during the second COVID-19 pandemic wave in Switzerland that this study provides, some limitations are worth mentioning. Of positive note is that we were able to assess longitudinally the use of coping strategies and depressive symptoms; however, we did not have pre-COVID-19 data. Therefore, we were not able to test whether the use of coping strategies or depressive symptoms had changed regarding pre-pandemic levels. Consequently, we cannot make long-term comparisons, which would have further complemented our findings. Nevertheless, in comparison to a large representative Swiss sample [49] using the same depression measure before the pandemic, our sample had clearly more depressive symptoms, speaking to the negative impact of the COVID-19 pandemic. Another note of caution refers to the assessment approach. Specifically, we collected data using an online platform which was easily accessible to anyone familiar with the internet, but less accessible for older adults lacking computer skills. Therefore, our findings regarding the representativeness of the Swiss older aged general population may be limited. Lastly, the problem-centered coping strategies assessed in this study focused on actively seeking functional or emotional support, as more relevant to the COVID-19 pandemic and its constraints. However, we lack the assessment of more specific problem-oriented coping strategies which may also have changed during this period and, therefore, cannot compare them with self-centered strategies or investigate how they may have been related to changes in depressive symptoms.

## Conclusions

This study provided a better understanding of the changes in the use of coping strategies during the second pandemic wave in Switzerland and their associations to level and change in depressive symptoms. In their effort to adapt to constantly changing pandemic developments and governmental regulations, including social-distancing measures, individuals developed more depressive symptoms and tried out different coping strategies, revealing a gap between the need for coping and the availability or utility of coping strategies. The strategies that were associated with feeling less depressed were self-centered coping strategies, aiming at reevaluating the COVID-19 situation as more positive. However, younger individuals were unable to frequently use this strategy, putting them at risk of developing more depressive symptoms. In unprecedented and unknown situations like the COVID-19 pandemic, individuals try to adjust by employing different coping strategies, without always succeeding, which is increasing the danger for mental health issues, as shown by this study. While our findings add to the understanding of how individuals adapt their coping response in stressful contexts such as the COVID-19 pandemic in order to maintain mental health, more work is needed to better support individuals in comparable future crisis situations.

## Electronic supplementary material

Below is the link to the electronic supplementary material.

Supplementary Material 1

## Data Availability

Data will become available upon acceptance of the article and the authors will provide the link to the data repository. The datasets generated and/or analysed during the current study will be available in the OSF repository with a DOI number. For the review process we provide the following link: https://osf.io/j4kub/?view_only=b01ae1d0a60f446982a0109814c4a8d3.

## Abbreviations

H: Hypothesis
MDCI: Multi-dimensional coping inventory
CES-D scale: Center for epidemiological studies depression scale
LGM: Latent growth-curve models
FIML: Full information maximum likelihood
CFI: Comparative fit index
TLI: Tucker lewis index
SRMR: Standardized root mean square residual
RMSEA: Root mean square error of approximation
AIC: Akaike’s information criterion
-2LL: -2 log likelihood
ICC: Intraclass correlation coefficient
CI: Confidence intervals
SD: Standard deviation
