# Supplementary material for: Development of depressive symptoms during the COVID-19 crisis: the role of coping strategies and their change
Source: BMC Psychol. 2025 Mar 14;13:250. doi: 10.1186/s40359-025-02406-8 (PMC11907944; doi:10.1186/s40359-025-02406-8)
Supplement: Supplementary file 1 — Supplementary Material 1 [file 40359_2025_2406_MOESM1_ESM.docx]

|  | **Depression**  **With mean and change in seeking functional and emotional support separately** | | | **Depression**  **With mean and change in seeking functional support only** | | | **Depression**  **With mean and change in seeking emotional support only** | | |
| --- | --- | --- | --- | --- | --- | --- | --- | --- | --- |
| *Predictors* | *Estimates* | *CI* | *p* | *Estimates* | *CI* | *p* | *Estimates* | *CI* | *p* |
| Time | 0.07 | 0.05 – 0.09 | **<0.001** | 0.07 | 0.05 – 0.09 | **<0.001** | 0.07 | 0.05 – 0.09 | **<0.001** |
| Age | -0.00 | -0.01 – -0.00 | **0.012** | -0.00 | -0.01 – -0.00 | **0.009** | -0.00 | -0.01 – -0.00 | **0.016** |
| Gender (Women = 1) | 0.08 | 0.01 – 0.15 | **0.030** | 0.09 | 0.02 – 0.15 | **0.016** | 0.08 | 0.00 – 0.15 | **0.037** |
| Married (Yes = 1) | -0.01 | -0.11 – 0.10 | 0.889 | -0.01 | -0.11 – 0.10 | 0.880 | -0.00 | -0.11 – 0.10 | 0.964 |
| Living alone (Yes =1) | 0.13 | 0.03 – 0.23 | **0.009** | 0.13 | 0.03 – 0.23 | **0.009** | 0.15 | 0.05 – 0.25 | **0.003** |
| Employed (Yes =1) | -0.18 | -0.26 – -0.10 | **<0.001** | -0.18 | -0.26 – -0.10 | **<0.001** | -0.19 | -0.26 – -0.11 | **<0.001** |
| Education years | 0.01 | -0.00 – 0.02 | 0.105 | 0.01 | -0.00 – 0.02 | 0.091 | 0.01 | -0.00 – 0.02 | 0.092 |
| Seek functional support (mean) | -0.12 | -0.18 – -0.06 | **<0.001** | -0.09 | -0.13 – -0.06 | **<0.001** |  |  |  |
| Seek emotional support (mean) | 0.04 | -0.02 – 0.10 | 0.206 |  |  |  | -0.06 | -0.09 – -0.02 | **0.003** |
| Positive reappraisal (mean) | -0.22 | -0.26 – -0.17 | **<0.001** | -0.21 | -0.26 – -0.17 | **<0.001** | -0.22 | -0.27 – -0.18 | **<0.001** |
| Acceptance (mean) | -0.05 | -0.10 – 0.01 | 0.086 | -0.05 | -0.10 – 0.01 | 0.096 | -0.05 | -0.11 – 0.00 | 0.069 |
| Self-distracting (mean) | 0.14 | 0.10 – 0.18 | **<0.001** | 0.14 | 0.10 – 0.18 | **<0.001** | 0.14 | 0.10 – 0.18 | **<0.001** |
| Seek functional support (change) | -0.05 | -0.09 – 0.00 | 0.057 | -0.06 | -0.10 – -0.02 | **0.007** |  |  |  |
| Seek emotional support (change) | -0.03 | -0.08 – 0.01 | 0.188 |  |  |  | -0.05 | -0.09 – -0.01 | **0.027** |
| Positive reappraisal (change) | -0.23 | -0.33 – -0.12 | **<0.001** | -0.23 | -0.34 – -0.12 | **<0.001** | -0.22 | -0.32 – -0.11 | **<0.001** |
| Acceptance (change) | 0.02 | -0.02 – 0.07 | 0.276 | 0.02 | -0.02 – 0.06 | 0.328 | 0.02 | -0.02 – 0.07 | 0.308 |
| Self-distracting (change) | 0.06 | 0.02 – 0.10 | **0.002** | 0.06 | 0.02 – 0.09 | **0.002** | 0.06 | 0.03 – 0.10 | **0.001** |
| Age * Positive reappraisal (change) | 0.00 | 0.00 – 0.01 | **0.022** | 0.00 | 0.00 – 0.01 | **0.020** | 0.00 | 0.00 – 0.01 | **0.033** |
| Residual Variance | 0.08 | | | 0.09 | | | 0.09 | | |
| Intercept | 0.13 _ID_ | | | 0.13 _ID_ | | | 0.13 _ID_ | | |
| Seeking support slope | 0.03 _ID.sfs_dev_ | | | 0.03 _ID.sfs_dev_ | | | 0.02 _ID.ses_dev_ | | |
| Self-distracting slope | 0.04 _ID.dst_dev_ | | | 0.04 _ID.dst_dev_ | | | 0.05 _ID.dst_dev_ | | |
| Residual Variance | -0.26 | | | -0.27 | | | -0.50 | | |
| Intercept | 0.17 | | | 0.17 | | | 0.19 | | |
| ICC | 0.63 | | | 0.63 | | | 0.63 | | |
| N | 696 _ID_ | | | 696 _ID_ | | | 696 _ID_ | | |
| Observations | 1762 | | | 1762 | | | 1762 | | |
| Marginal R^2^ / Conditional R^2^ | 0.310 / 0.742 | | | 0.306 / 0.742 | | | 0.297 / 0.741 | | |
